# Typhoid Mortality

**Published:** 1912-02

**Authors:** 


### Typhoid Mortality. What May Be Done.

We appropriate the following table from the Canada *Lancet.*

THE ANNUAL DEATH RATE FROM TYPHOID FEVER PER MILLION OF THE POPULATION.

| | Average 1901-1904. | Average 1905-1908. | Per Cent. of Decrease. |
|---|---|---|---|
| Spain | 459 | 362 | 21 |
| U. S. (Reg. Area) | 332 | 288 | 13 |
| Italy | 358 | 283 | 20 |
| Austria | 198 | 156 | 21 |
| Servia | 808 | 147 | 81 |
| Belgium | 175 | 122 | 30 |
| Ireland | 134 | 91 | 32 |
| England and Wales | 118 | 80 | 31 |
| Scotland | 122 | 74 | 38 |
| Netherlands | 87 | 72 | 16 |
| Prussia | 91 | 61 | 32 |
| German Empire | 75 | 53 | 29 |
| Switzerland | 65 | 46 | 28 |

It is only fair to observe that Switzerland has advantages in regard to water supply which are almost without parallel, most of the country being within easy piping distance of what is practically distilled water, precipitated on an almost uninhabitable region and deliverable by gravity sufficiently cool for drinking at any time of the year. But it is not pleasant to think that the United States has next to the worst record of the civilized world and that, in spite of its natural advantages, it has four times the mortality of Holland, overcrowded, water-soaked and with little dependence on gravity either for obtaining water or carrying off sewage. We know what to do, let us do it.

